# Effects of hypoxia/hyperoxia exposure on immune function – results from a spacecraft-relevant hypobaric chamber study

**DOI:** 10.3389/fphys.2025.1637834

**Published:** 2025-09-09

**Authors:** Brian Crucian, Douglass M. Diak, Alejandro Garbino, Cody Gutierrez, Sara Bustos-Lopez, Audrie Colorado, Millennia Young, Scott M. Smith, Sara R. Zwart, Thomas M. Oswald, Monica Y. Hew-Yang, Patrick Estep, Karina Marshall-Goebel, Satish Mehta

**Affiliations:** ^1^ NASA Johnson Space Center, Human Health and Performance Directorate, Houston, TX, United States; ^2^ Aegis Aerospace, Houston, TX, United States; ^3^ Geocontrol Systems, Houston, TX, United States; ^4^ JES Tech, Houston, TX, United States; ^5^ KBR, Houston, TX, United States; ^6^ Aerospace Medicine Division, School of Public and Population Health, University of Texas Medical Branch, Galveston, TX, United States

**Keywords:** immunity, spaceflight, hypoxia, hyperoxia, extravehicular activity

## Abstract

**Introduction:**

Although the International Space Station provides a normoxic environment, deep space missions are expected to leverage a hypobaric, mildly hypoxic living environment to facilitate frequent extravehicular activities EVAs, aka spacewalks. Although hypoxia may be experienced terrestrially, it will be atypical for human physiology to live in hypobaric/hypoxic conditions yet frequently experience hyperoxic stress due to EVAs. It is well established that hypoxia induces dysregulation of the human immune system, in generally a sensitized/proinflammatory fashion. This is primarily evidenced from studies of individuals living at altitude.

**Methods:**

To ascertain the effect of hypoxic/hyperoxic shifts on immunity, a series of 11 days hypobaric chamber studies were conducted at the Johnson Space Center. The living environment consisted of 8.2–9.6psi/28.5%–34% oxygen, and there were several simulated EVAs which were performed under hypobaric/hyperoxic conditions consisting of 4.3psi/85%–95% oxygen. For the current sub-study, biosamples were collected before and after simulated EVAs to ascertain the effects of hypoxia, decompression and hyperoxic stress on immunity. The sub-study consisted of 3 chamber tests, 23 total subjects.

**Results:**

Shifts in leukocyte distribution, function, and plasma cytokine concentrations were associated with atmospheric shifts, primarily after the hypobaric/hyperoxic EVA activities. Astronauts already experience immune system dysregulation due to microgravity, stress, and other mission influences.

**Discussion:**

These data indicate that, similar to living at high altitude, altered atmosphere exposure in a pressurized vehicle environment may dysregulate human immunity which may be exacerbated by EVAs. The additive effects of hypoxia, in concert with other spaceflight mission variables, on clinical risks for astronauts must be better characterized to enable future exploration class space missions.

## Background

Orbital spaceflight onboard the Space Shuttle and the International Space Station (ISS) were operated in a normoxic, sea-level atmosphere, i.e., 14.7 psi and 21% oxygen. Spacewalks, or extravehicular activities (EVAs) are required to be performed in the lower pressure, mildly hyperoxic atmosphere, of a spacesuit. Typically, the EVA suit atmosphere was 4.3 psi and >95% oxygen. To avoid decompression sickness, the EVA required a denitrogenation (oxygen prebreathe) protocol that took several hours (Brady and Polk, 2011).

Future deep space exploration missions, particularly those on planetary surfaces, will entail a higher frequency of EVAs than on missions to low Earth orbit. Also, it is not desirous to require a prolonged oxygen prebreathe prior to the EVA. Instead, quick exit out of the habitat or pressurized rover would be beneficial, to enable frequent EVAs during exploration missions. The current concept of operations under consideration by the Artemis Program is an ambient vehicle/habitat ‘exploration atmosphere’ of 8.2 psi and 34% O2 (piO2 128 mmHg) (Norcross et al., 2013). This would allow for a shorter oxygen prebreathe (∼20 min), greatly shortening the logistics before exiting the vehicle for an EVA and greatly facilitating exploration operations. EVA suit atmosphere would still be hyperoxic (piO2 222 mmHg). Deep space explorers would therefore be frequently cycling between hypoxic and hyperoxic conditions. There is evidence that hyperoxia exposures influenced brain perfusion, cognition, and EEG activity (Damato et al., 2020; Damato et al., 2022b). There is very little existing research data regarding the effects of such atmospheric cycling on physiology.

Spaceflight mission stressors other than altered atmosphere, such as microgravity, circadian misalignment and physiologic stress, result in the persistent dysregulation of the human immune system (Krittanawong et al., 2022; Rooney et al., 2019). The phenomenon is characterized by reductions in T and NK cell function, altered cytokine profiles, and the reactivation of latent herpesviruses (Crucian et al., 2015; Bigley, 2019; Crucian et al., 2014; Mehta et al., 2017; Rooney et al., 2019). While these alterations have not caused widespread clinical issues, some crewmembers experience immune-related adverse events, including manifestations of symptomatic herpes viral reactivation, allergy, and respiratory distress (Crucian et al., 2016a IJGM). Three case reports have been published of astronauts regarding clinically relevant immune compromise, and dermatitis on orbit, two of which confirmed the involvement of latent herpesvirus reactivation (Crucian et al., 2016b; Mehta et al., 2022; Mehta et al., 2024). One ISS crewmember experienced zoster (laboratory confirmed by skin swab) on orbit, and the outbreak was correlated with peaks in both immune dysfunction and stress hormone concentrations (Mehta et al., 2024). Another case correlated dermatitis flares with operational stressors (Crucian et al., 2016a). In a third case, virus ‘evolution’ onboard ISS, defined as increasing micro-variants, was documented (Mehta et al., 2022). Such cases are important as they demonstrate that the immune dysregulation in astronauts can progress to operationally-impactful adverse clinical events during flight. It is reasonable to hypothesize that the immune dysregulation observed aboard ISS missions will intensify during longer missions in deep space, thereby placing crewmembers at elevated clinical risk (Crucian and Sams, 2009).

Other environments, such as saturation diving and Antarctic winterover, share many isolation and mission stressors with spaceflight, including station lifestyle, extreme environment, and 6–12 months deployments. Saturation diving can also include frequent changes to atmospheric pressure, extended duration living in altered atmospheric conditions, and challenges with humidity and sea life induced contamination (Brubakk et al., 2014). Findings from immune studies at Concordia Antarctic Station were surprisingly discordant from spaceflight findings. Whereas spaceflight generally results in a suppression of leukocyte function, at Concordia (innate) cellular function was generally sensitized, with functional responses elevated (Feuerecker et al., 2014; Feuerecker et al., 2019). The only confounding variable thought best to be responsible for this sensitization was the persistent hypobaric hypoxia as Antarctica is a mountain, and Concordia Station sits at 10,606 feet in elevation (piO2 ∼97 mmHg).

Hypoxia is known to regulate and/or influence immunity, inflammation, and physiology in a generally stimulatory fashion, as reviewed by Taylor et al. (Taylor and Colgan, 2017; Damato et al., 2022a). Hypoxia can influence immune cellular functional capacity (e.g., proliferation, development), or can function primarily via transcriptional changes mediated by hypoxia-inducible factor. While there is variability among altitude/hypoxia studies, generally, effects on immunity are stimulatory, and pro-inflammatory (Kubo et al., 1998; Hartmann et al., 2000; Coussons-Read et al., 2002; Facco et al., 2005; Ermolao et al., 2009), while other reports suggest lymphocytosis, or HPA axis mediated immune suppression (Siqués et al., 2007; Chen et al., 2012; Oliver et al., 2013; Kleessen et al., 2005) or innate cellular migration and inflammation (Pahl et al., 2006). Hypoxia also specifically influences anti-tumor immunity (Arnaiz and Harris, 2022). Physical activity or exercise at altitude in a hypoxic environment had differential influences (de Aquino Lemos et al., 2013; Mazzeo, 2005; Tiollier et al., 2005; Choukèr et al., 2005). A recent study suggests that normobaric hypoxia training and increased erythropoiesis impacts iron metabolism, which can challenge maintenance of immune function (Nolte et al., 2025). The effects of hypoxia on immunity appear highly biased, meaning some aspects of inflammation/immunity may be suppressed, whereas others may be heightened or sensitized. This effect may be exploited as a treatment for specific disease states related to an alteration in immune bias. For example, hypoxia can be beneficial for asthma allergy, or eczema, which are all considered to be ‘T_H_2’ diseases, or certain tumors resulting in intra-tumor localized hypoxia (Schultze-Werninghaus, 2008; Engst and Vocks, 2000; Hatfield et al., 2015).

Current denitrogenation prebreathe protocols performed prior to conducting a spacewalk on the ISS, from a normoxic vehicle to a hypobaric spacesuit, last about 4 h. EVAs will be much more frequent during upcoming planetary exploration. To optimize time spent exploring the Lunar surface and minimize prebreathe durations while maintaining a safe risk posture for decompression sickness risk prevention, an ‘exploration atmosphere’ is being planned for the Artemis program, where the vehicle will be at 8.2 psi and 34% oxygen (or similar), allowing for significantly shorter prebreathe durations prior to EVA decompression. There are varied literature reporting immune dysregulation associated with hypoxia, primarily derived from studies of people living at altitude, as well as decompression stress.

Immune dysregulation has also been well documented to be associated with other spaceflight stressors, such as microgravity, isolation, circadian misalignment, or altered diet. This has been observed in studies of astronauts during flight, as an amalgamation of all these mission stressors, and also in ground-space-analog studies, where such variables may be studied individually. Given that deep space missions will likely be conducted in a uniquely hypobaric/hypoxic environment with intermittent hypobaric/mildly hyperoxic EVAs, the current study was conducted in a hypobaric chamber housed at the Johnson Space Center. Subjects lived in the chamber for 11 days in a mildly hypoxic environment, and during several mission days participated in hyperoxic simulated ‘EVAs’. This operational scenario is a high-fidelity representation of the atmosphere planned for deep space missions. Biosampling was conducted on several days, both before and after EVA. The objective was to allow a determination of the effects of both hypoxia and hyperoxia (and cycling therein) on immunity, inflammation, and latent herpesvirus reactivation.

## Methods

### Subjects

Subjects were healthy volunteers, medically screened by the JSC Test Subject Facility, consented and enrolled to participate. Subject demographic information is presented in Table 1. For this study, there were three complete 11-day chamber missions, each with 8 participating subjects. Six were full subjects, participating in all simulated EVA activities, and the remaining two were ‘Doppler technicians’, involved in the collection of other study data during the EVAs. Of the 24 total mission participants, four individuals repeated in a second mission, therefore 20 individuals participated in one or more missions. Of the 20 participating individuals, 10 were male and 10 were female. The Doppler technicians lived in the chamber but did not participate in the physical activities associated with EVA. The Doppler Technicians did experience all altered atmosphere cycles, isolation, diet, circadian misalignment, and all other ‘chamber variables’, and performed Doppler imaging as part of their work requirement. One subject dropped during the third mission, resulting in a final study ‘n’ of 23 subjects.

**TABLE 1:** Complete blood count parameters and peripheral leukocyte distribution by flow cytometry (data are means ± SEM).

Variable	BDC AM	MD3 a.m.	MD3 p.m.	MD7 a.m.	MD7 p.m.	MD10 a.m.
(Complete Blood Count Parameters)						
WBC (10^3/mm3)	5.7 ± 0.20	5.5 ± 0.26	**8.6 ± 0.46** ^a^ ^,^ ^b^	5.7 ± 0.27	**7.1 ± 0.47** ^a^ ^,^ ^b^	5.3 ± 0.30
Neutrophils (%)	52 ± 1.5	50 ± 1.3	**59 ± 1.8** ^a^ ^,^ ^b^	**46 ± 1.5** ^a^	**55 ± 1.7** ^b^	**46 ± 1.7** ^a^
Lymphocytes (%)	37 ± 1.4	39 ± 1.2	**32 ± 1.8** ^a^ ^,^ ^b^	**41 ± 1.6** ^a^	**35 ± 1.6** ^b^	42 ± 1.9
Monocytes (%)	7.0 ± 0.32	7.4 ± 0.45	**6.3 ± 0.32** ^a^ ^,^ ^b^	**7.8 ± 0.47** ^a^	**6.4 ± 0.36** ^a^ ^,^ ^b^	7.5 ± 0.41
Eosinophils (%)	3.1 ± 0.34	2.9 ± 0.31	**1.4 ± 0.18** ^a^ ^,^ ^b^	3.2 ± 0.43	**1.9 ± 0.34** ^a^ ^,^ ^b^	3.5 ± 0.49
Basophils (%)	0.69 ± 0.059	0.66 ± 0.068	**0.55 ± 0.050** ^a^ ^,^ ^b^	0.80 ± 0.085	**0.60 ± 0.062** ^b^	0.71 ± 0.067
RBC (10^6/mm3)	4.9 ± 0.11	5.0 ± 0.13	5.1 ± 0.13	**5.3 ± 0.097** ^a^	**5.0 ± 0.13** ^b^	**5.4 ± 0.11** ^a^
Hemoglobin (g/dl)	14 ± 0.27	14 ± 0.31	15 ± 0.31	**15 ± 0.22** ^a^	**14 ± 0.31** ^b^	**16 ± 0.30** ^a^
Hematocrit (%)	41 ± 0.70	41 ± 0.88	42 ± 0.83	**43 ± 0.63** ^a^	**41 ± 0.90** ^b^	**44 ± 0.83** ^a^
MCV (fl)	82.8 ± 1.1	**81.9 ± 1.0** ^a^	**82.7 ± 1.0** ^a^ ^,^ ^b^	**83.1 ± 1.0** ^a^	**84.3 ± 0.98** ^b^	83.7 ± 1.0
MCH (pg)	29 ± 0.43	29 ± 0.42	29 ± 0.43	29 ± 0.43	29 ± 0.43	29 ± 0.42
MCHC (g/dl)	35.0 ± 0.23	**35.2 ± 0.24** ^a^	**35.6 ± 0.20** ^a^ ^,^ ^b^	**35.4 ± 0.23** ^a^	35.0 ± 0.28	35.7 ± 0.25
RDW (%)	13 ± 0.12	**13 ± 0.12** ^a^	**12 ± 0.12** ^a^ ^,^ ^b^	13 ± 0.12	13 ± 0.13	13 ± 0.12
Platelet (10^3/mm3)	67 ± 7.2	91 ± 12	67 ± 7.9	65 ± 9.3	69 ± 14	**100 ± 12** ^a^
(Leukocyte Distribution by Flow Cytometry in %) (data are means ± SEM)						
T cells	70 ± 1.2	68 ± 2.0	62 ± 4.6	**74 ± 1.3** ^a^	74 ± 1.9	70 ± 1.1
B cells	8.6 ± 0.56	8.2 ± 0.77	**10 ± 1.0** ^a^ ^,^ ^b^	**7.1 ± 0.69** ^a^	**9.4 ± 1.1** ^b^	8.1 ± 0.80
NK Cells	14 ± 1.1	13 ± 1.1	**6.8 ± 0.72** ^a^ ^,^ ^b^	13 ± 1.2	**9.3 ± 1.3** ^a^ ^,^ ^b^	14 ± 1.1
NK-T Cells	2.9 ± 0.40	2.5 ± 0.46	**1.6 ± 0.24** ^a^ ^,^ ^b^	2.1 ± 0.49	2.6 ± 0.51	2.5 ± 0.43
Helper T cells	62.7 ± 1.9	62.6 ± 1.9	63.4 ± 1.6	65.5 ± 2.3	62.2 ± 1.8	62.7 ± 1.6
Cytotoxic T cells	27.5 ± 1.6	28.4 ± 1.7	**27.4 ± 1.4** ^b^	26.2 ± 2.2	**29.3 ± 1.6** ^b^	28.0 ± 1.6
CD4+ True Naïve	50 ± 2.8	50 ± 2.8	**46 ± 3.0** ^a^ ^,^ ^b^	51 ± 3.5	47 ± 3.2	52 ± 2.7
CD4+ Central Memory	36 ± 2.0	**33 ± 2.0** ^a^	**32 ± 2.1** ^a^	**30 ± 2.3** ^a^	**35 ± 2.1** ^b^	**33 ± 2.0** ^a^
CD4+ Term. Differentiated	1.1 ± 0.47	2.0 ± 0.67	3.2 ± 1.1	6.7 ± 4.9	2.4 ± 1.1	1.8 ± 1.0
CD4+ Effector Memory	13 ± 1.3	15 ± 1.8	**18 ± 2.3** ^a^	12 ± 1.3	**16 ± 1.8** ^b^	12 ± 1.2
CD8+ True Naïve	52 ± 3.1	52 ± 3.2	**57 ± 2.8** ^a^ ^,^ ^b^	**56 ± 3.3** ^a^	55 ± 3.2	54 ± 3.0
CD8+ Central Memory	12 ± 1.2	12 ± 1.1	12 ± 0.92	**10 ± 0.93** ^a^	**12 ± 0.93** ^b^	11 ± 1.1
CD8+ Term. Differentiated	13 ± 2.6	13 ± 2.5	**9.9 ± 1.8** ^b^	14 ± 2.5	12 ± 2.6	13 ± 2.6
CD8+ Effector Memory	23 ± 2.1	23 ± 2.1	22 ± 2.1	**20 ± 2.0** ^a^	21 ± 1.9	21 ± 2.1
Naïve B cells	69 ± 2.2	69 ± 2.1	**55 ± 4.3** ^a^ ^,^ ^b^	65 ± 2.5	68 ± 2.5	68 ± 2.7
Non-switched Memory B Cells	5.8 ± 0.90	5.2 ± 0.88	**9.1 ± 1.6** ^b^	9.5 ± 1.7	8.1 ± 1.5	5.6 ± 1.0
Switched Memory B Cells	10 ± 1.2	9.0 ± 0.97	8.5 ± 1.2	11 ± 1.0	9.4 ± 1.3	8.9 ± 0.79

^a^p<0.05 compared to BDC.

^b^p<0.05 p.m., compared to AM (same day).

### Samples

**FIGURE 1:**
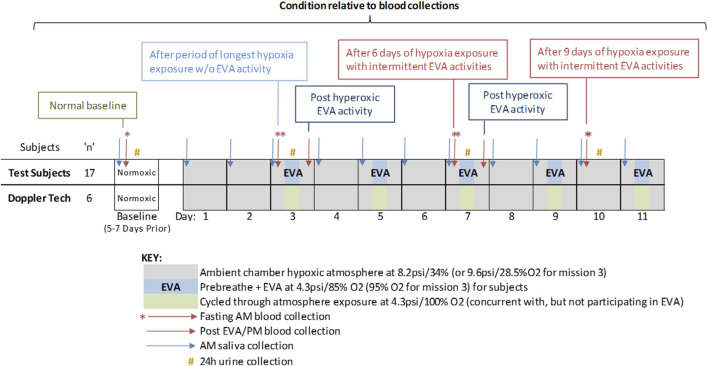
Study design including duration, atmosphere composition, EVA days, and biosample collections. The study was designed to assess the effects of mild hypoxia (baseline and Day 3 – AM); hyperoxic EVA activity (Day 3 and 7, post EVA), and living in mild hypoxia interspersed with hyperoxic challenging EVAs (Day 7 and 10, a.m.). Doppler technicians live inside the chamber and also experienced the hypoxia conditions during EVAs, but did not participate in the physical activities.

Sampling schedule is described in Figure 1. In brief, samples consisted of 1.0 mL saliva, sampled early morning on all collection days, which occurred at baseline (5–7 days prior to entering the chamber) and each mission day, for stress hormone and latent virus DNA measurements. Saliva was collected via passive drool, and frozen immediately until batch analysis. Ambient blood was collected in a 6.0 mL heparin anticoagulated monovette at baseline and 5 times inside the chamber during the mission. The mission blood collections were scheduled to allow some interpretation of the effects of hypoxia, hyperoxic EVA and decompression stress, and cycling between the two as indicated (Figure 1). Urine samples were collected over a 24 h period during baseline data collection (BDC), and on mission days (MD) 3, 7, and 10. A 5 mL aliquot was removed from the 24 h pool and frozen at −80°C until batch processing.

### Chamber and atmosphere

**FIGURE 2:**
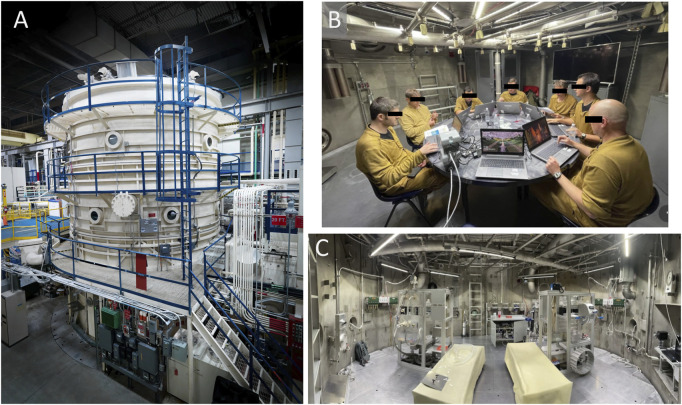
**(A)** Image of the hypobaric chamber at the NASA Johnson Space Center; **(B)** crewmembers inside the chamber during the study; **(C)** chamber EVA simulation stations. Photo credits to NASA.

A livable 20-foot diameter hypobaric chamber was outfitted with furniture and equipment to support multiple subjects living in the chamber for 11 days (Figure 2). This was a program level evaluation for NASA with multiple objectives. Prior to depressing the chamber to the exploration atmosphere, subjects underwent a 180 min 100% Oxygen prebreathe. Thereafter, they lived in the chamber for 11 days at the exploration atmosphere: 8.2psi/34%O2 for missions 1 and 2 and 9.6psi/28.5%O2 for mission 3. The atmosphere was changed for mission 3 to address a NASA desire to explore options for programs and vehicles that could not readily achieve >30%O2 due to flammability limitations. Simulated EVAs were conducted on MD 3, 5, 7, 9, and 11. Prior to simulated EVAs, subjects underwent a 85% O2 - 15%N2 (95%O2 - 5% N2 when coming from the 9.6psi atmosphere) prebreathe and the chamber pressure was then reduced to 4.3psi to simulate spacesuit pressure for the simulated EVA. The simulated EVAs were conducted shirt sleeve (not in a spacesuit) and subjects conducted various activities representative of the metabolic workloads expected during Lunar surface EVAs for 6 h with a targeted average metabolic workload of ∼40% of VO2max. Simulated EVAs and associated physical activities were identical across the simulated EVAs on MD3, 5, 7, 9, and 11.

The Doppler technicians lived at the same ambient chamber atmosphere, but during the EVA activities were masked to 100% oxygen as a protective measure and were tasked with monitoring venous gas emboli in the active subjects; thus did not partake in the metabolic load of the simulated EVA activities (remained at a resting metabolic rate) but underwent the same decompression profile. After each EVA, the hypobaric chamber was re-established to the exploration atmosphere (8.2 psi/34% O2 or 9.6psi/28.5%O2) prior to removing their breathing masks.

**FIGURE 3:**
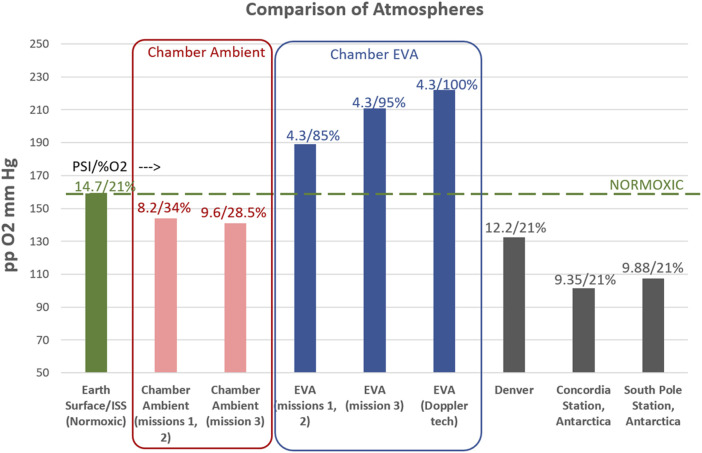
Comparison of Atmospheres between Earth (sea level), International Space Station (ISS), chamber conditions for this study, and relevant terrestrial locations/altitudes. A comparison of the partial pressure of O2, in mmHg, is represented (Y-axis), while the atmospheric pressure (psi) and percent oxygen are indicated above each data point. Note that for the chamber conditions, the ambient living atmosphere is mildly hypoxic for all three missions. Mission 3 had a slightly different atmosphere, but a similar level of mild hypoxia. The EVA conditions were hyperoxic as indicated for all missions. During EVA, regular subjects experienced the 4.3psi/85%–95% O2 atmosphere and participated in physical activities associated with spacewalks, where the Doppler technicians were masked to 100% O2 and did not participate in the physical activities. As no differences were seen between the missions, or the EVA/Doppler Technician groups, data remain grouped as a single (8 subjects enrolled per mission, 23 completed subjects total) cohort.

As no differences were observed between mission 3 and missions 1,2 (data not shown), the hypoxia exposure is similar (mission 3: pp O2 141 mmHg; missions 1, 2: pp O2 144 mmHg) and the hypobaric/hyperoxic EVA exposures were similar (mission 3: 4.3psi, 95% O2, missions 1, 2: 4.3psi, 85% O2) (Figure 3), for purposes of this product and to increase statistical power, the three chamber missions were grouped as a single cohort. Similarly, no differences were observed between the prime EVA subjects and the Doppler technicians, who during the EVA were on a slightly different hyperoxic atmosphere (Figure 3). Therefore, for purposes of these analyses all 23 subject across 3 missions were analyzed as a single cohort.

### Complete blood count

A complete blood count, WBC, bulk leukocyte subsets via potentiometric measurement (no positive identification via surface marker expression), and RBC parameters was performed using standard techniques using a Sysmex XM-10 (Kobe, Hyogo, Japan) hematology analyzer according to the manufacturer’s instructions.

### Flow cytometry/leukocyte subsets

A comprehensive 5-color flow cytometry antibody matrix, up to 10 colors per analysis tube, was formulated for peripheral blood immunophenotype analysis. Fluorescent labeled antibodies were obtained from Cytex (Amsterdam The Netherlands). The specific fluorescent antibody matrix setup, relevant leukocyte subsets identified, as well as blood sample staining and lysis protocols, and flow cytometry analysis were set up and performed on a Beckman Coulter ‘Gallios’ flow cytometer as previously described (Crucian et al., 2013). Specific antibodies utilized were as follows:

Tube A: IgD (FITC), CD56 (PE), CD19 (PC5), CD14 (PC7), CD45 (APC), CD27 (APC-Cy7), CD3 (V500).

Tube B: CD57 (FITC), CD62L (PE), CD28 (PC5), CCR7 (PC7), CD45RA (APC), CD4 (APC-Cy7), CD8 (Pac Blue), CD3 (V500).

This panel assessed the major leukocyte and lymphocyte subsets, T-cell subsets, memory/naïve and central memory T-cell subsets, and constitutively activated T-cell percentages. Whole blood, without any artificial cellular enhancement or purification, was stained for analysis.

### Plasma cytokine concentrations (30 plex)

Plasma was aliquoted from 7.5 mL Lithium Heparin Sarstedt blood tube after centrifugation at 974 RCF for 15 min. Plasma samples were vortexed and centrifuged at 10,621 X RCF for 10 min. Samples were ran neat. Cytokine concentration was measured using a Milliplex Human Cytokine/Chemokine MAGNETIC BEAD Premixed 30 Plex Kit (Millipore Sigma, Chicago, IL, United States) according to manufacturer’s instructions. Samples were analyzed on a Luminex Magpix instrument with xMAP INTELLIFLEX software (Luminex, Austin, TX, United States). The 30 cytokines analyzed were IL-1a, IL-1b, TNFa, IL-6, IL1ra, IL-8, IL-2, IFNg, IL-4, IL-5, IL-10, IL-17a, CCL2/MCP-1, CCL3/MIP-1 alpha, CCL4/MIP-1 beta, CCL5/RANTES, GCSF, GM-CSF, VEGF, IL-3, IL-7, EGF, Eotaxin, IL-12 (p40), IL-12 (p70), IL-15, IP-10, IL-13, IFN-a2 and TNFb.

### Leukocyte function (mitogen stimulated cytokine profiles)

Whole blood culture setup, mitogen stimulation, and maintenance was performed as previously described (Feuerecker et al., 2019). Supernatant from cell cultures were collected after 48 h of culture time. Samples were vortexed and centrifuged at 10,621 X RCF for 10 min. Samples were diluted 3:1 with provided Assay Buffer. Cytokine concentration was measured using a MILLIPLEX MAP Human High Sensitivity T Cell Magnetic Bead 13 Plex Panel assay (Millipore Sigma, Chicago, IL, United States) according to manufacturer’s instructions. Samples were analyzed on a Luminex Magpix instrument with xMAP INTELLIFLEX software (Luminex, Austin, TX, United States). The 13 cytokines analyzed were GMCSF, IFNy, IL-10, IL-12 (p70), IL-13, IL-1B, IL-2, IL-4, IL-5, IL-6, IL-7, IL-8 and TNFa.

### Saliva cortisol

Frozen saliva samples were thawed at room temperature, vortexed, and up to 1 mL was aliquoted for analysis. All samples were centrifuged at 18,000 x g for 20 min. Up to 800 uL of supernatant was removed and saved for salivary cortisol analysis. Analysis of salivary cortisol from the saliva supernatant was performed using Salimetrics Expanded Range High Sensitivity Salivary Cortisol Enzyme Immunoassay Kit (State College, PA; Cat. No. 1–3,002) per manufacturer’s instructions.

### Saliva latent herpesvirus DNA

Following saliva separation for cortisol analysis, the remaining 200ul (cell pellet) was DNA extracted using the QIAamp DNA Blood Mini Kit (Qiagen, Hilden, Germany; Cat. No. 51106), per manufacturer’s instructions. Viral loads (copies/mL saliva) for EBV, HSV-1, and VZV were determined for each sample by standard curve analysis using the QuantStudio 3 Real-Time PCR System (Thermo Fisher Scientific, Waltham, MA). Primers and probes for each virus have been previously published (Mehta et al., 2014; Mehta et al., 2022). DNA concentration was determined by Qubit 4 Fluorometer (Invitrogen, Thermo Fisher Scientific, Waltham, MA) using Invitrogen™ QuantiT™ Qubit™ dsDNA HS Assay Kit (Invitrogen, Thermo Fisher Scientific, Waltham, MA, Cat. No. Q32854) per the manufacturer’s instructions. PCR on GAPDH was used as a quality control. GAPDH primers and probe have been published previously (Cohrs et al., 2008).

### Urine latent herpesvirus DNA

Urine samples were then thawed, homogenized, and a 2 mL aliquot was removed for DNA extraction using Qiagen Viral RNA Mini Kit (Qiagen, Hilden, Germany, Cat. No. 52906). Briefly, 2 mL of urine was centrifuge at 14,000 rpm for 10 min and then 1860 µL of supernatant was removed leaving 140 µL urine and the cell pellet. DNA extraction on the cell pellet was performed according to the manufacturer’s instructions. DNA concentration was determined by Qubit 4 Fluorometer (Thermo Fisher Scientific, Waltham, MA) and CMV qPCR was conducted using the QuantStudio 7 Real-Time PCR System (Thermo Fisher Scientific, Waltham, MA). CMV primers and probes have been published previously (Mehta et al., 2024). PCR on GAPDH was used as a quality control. GAPDH primers and probe have been published previously.

### Statistical analysis

Mixed models were chosen for testing for flexibility in accounting for the repeated measures design and inherent imbalances across factors, like PM only being measured on MD3 and MD7. Each immune measure was analyzed using linear mixed-effects models defined in terms of the interaction of Mission Day (BDC, MD3, MD7, or MD10) and time (AM or PM). Random effects included the within-subject repeated measures (subject-specific intercepts), and the nesting of subjects within missions to fully address the data dependences. Additionally, models included robust standard errors to allow for non-homogenous variance across conditions. Marginal means were used for estimation and pairwise comparisons with simulation-method p-value adjustments. When the overall F-test was significant, pairwise comparisons were conducted to assess difference from baseline (BDC) for all other timepoints, and between AM and PM on MD3 and MD7. Analyses were conducted in SAS v9.4 using the GLIMMIX procedure and LSMEANS/LSMESTIMATE statements.

## Results

### CBC/peripheral leukocyte distribution

In mission, whole blood cells (WBCs) were significantly elevated after the EVA activities on MD3 and MD7, both in comparison to the BDC as well as to the AM timepoint of each day (Table 2). The post-EVA elevation in WBC was driven by an increase in the relative percentage of neutrophils which became significantly elevated after each EVA activity (Table 2). In what may be a rebound effect, the neutrophil percentage was significantly reduced on the AM samplings of MD7 and MD10 when compared to BDC. There was a concurrent opposite response in the percentage of lymphocytes, monocytes, eosinophils and basophils that tracked with the neutrophil changes (Table 2).

**TABLE 2:** Plasma cytokine concentrations (pg/mL) (data are means ± SEM).

Variable	BDC AM	MD3 a.m.	MD3 p.m.	MD7 a.m.	MD7 p.m.	MD10 a.m.
(Inflammatory Cytokines)						
IL-1a	51 ± 21	50 ± 19	54 ± 20	56 ± 22	55 ± 20	57 ± 22
IL-1B	7.4 ± 1.7	5.9 ± 0.65	7.0 ± 1.3	8.9 ± 2.7	8.6 ± 2.1	10 ± 3.6
IL-6	9.2 ± 4.1	7.5 ± 2.9	**13 ± 4.2** ^a^ ^,^ ^b^	9.9 ± 4.0	11 ± 4.2	11 ± 3.8
IL-8	29 ± 10	19 ± 5.3	21 ± 6.8	27 ± 7.8	25 ± 6.8	19 ± 6.2
TNFa	23 ± 2.3	23 ± 2.8	24 ± 2.6	25 ± 3.2	25 ± 3.5	28 ± 4.5
IL-12 (p40)	64 ± 9.3	64 ± 9.5	**70 ± 10** ^b^	69 ± 11	66 ± 8.9	74 ± 12
IL-12 (p70)	16 ± 4.6	17 ± 4.6	21 ± 6.7	24 ± 8.5	26 ± 11	31 ± 15
IL-13	65 ± 15	64 ± 13	68 ± 14	71 ± 17	68 ± 14	80 ± 22
IL-17 F	64 ± 16	61 ± 17	69 ± 20	59 ± 12	68 ± 19	71 ± 20
IL-18	61 ± 5.2	63 ± 4.5	**68 ± 5.7** ^b^	**69 ± 6.1** ^a^	68 ± 5.2	70 ± 6.6
IL-22	220 ± 63	230 ± 62	250 ± 63	240 ± 58	230 ± 65	**290 ± 75** ^a^
(Anti-Inflammatory Cytokines)						
IL-1RA	35 ± 5.9	36 ± 5.7	38 ± 6.3	**48 ± 9.2** ^a^	**49 ± 9.8** ^a^	51 ± 14
(Adaptive/Regulatory Cytokines)						
IFNy	150 ± 31	170 ± 37	160 ± 46	190 ± 38	180 ± 52	180 ± 57
IL-2	3.9 ± 0.89	3.9 ± 0.87	4.8 ± 1.4	5.4 ± 1.6	5.7 ± 2.1	6.7 ± 2.6
IL-4	200 ± 110	200 ± 100	220 ± 110	220 ± 120	220 ± 120	190 ± 110
IL-5	7.1 ± 2.3	6.9 ± 2.1	8.2 ± 2.6	8.2 ± 2.8	8.3 ± 2.4	8.7 ± 2.3
IL-10	24 ± 6.5	22 ± 5.8	25 ± 7.0	23 ± 6.2	24 ± 6.3	27 ± 7.5
IL-17E/IL-25	1,100 ± 180	1,100 ± 170	**980 ± 140** ^b^	1,200 ± 260	1,000 ± 170	1,200 ± 350
IL-17A	16 ± 3.7	17 ± 3.6	**21 ± 5.2** ^b^	20 ± 5.6	21 ± 6.8	25 ± 7.4
(Growth Factors)						
G-CSF	32 ± 4.8	28 ± 3.9	**42 ± 6.7** ^a^ ^,^ ^b^	42 ± 10	38 ± 7.3	28 ± 5.7
GM-CSF	22 ± 11	17 ± 8.5	19 ± 8.1	24 ± 16	27 ± 11	37 ± 24
VEGF-A	72 ± 13	75 ± 13	78 ± 18	88 ± 18	92 ± 24	94 ± 27
EGF	37 ± 5.5	35 ± 5.2	29 ± 4.0	**53 ± 8.0** ^a^	**33 ± 4.5** ^b^	31 ± 5.5
IL-3	1.3 ± 0.23	1.3 ± 0.23	1.3 ± 0.23	1.3 ± 0.23	1.3 ± 0.23	1.3 ± 0.23
IL-7	8.4 ± 3.8	9.2 ± 5.1	9.8 ± 5.4	8.2 ± 3.7	8.6 ± 3.7	9.3 ± 4.5
IL-15	9.7 ± 1.4	9.8 ± 1.8	**12 ± 2.6** ^b^	10 ± 2.1	11 ± 2.2	13 ± 3.1
PDGF-AA	3,500 ± 550	3,700 ± 760	**1,400 ± 160** ^a^ ^,^ ^b^	**4,600 ± 560** ^a^	**3,000 ± 660** ^b^	2,400 ± 210
PDGF-AB/BB	8,300 ± 940	8,500 ± 1,100	**4,800 ± 470** ^a^ ^,^ ^b^	**10,000 ± 990** ^a^	**7,300 ± 1,100** ^b^	6,500 ± 590
M-CSF	54 ± 7.5	48 ± 4.5	**42 ± 4.7** ^a^	54 ± 8.3	**42 ± 4.3** ^a^ ^,^ ^b^	50 ± 10
(Chemokines)						
MCP-1	340 ± 23	340 ± 22	**470 ± 27** ^a^ ^,^ ^b^	350 ± 19	410 ± 48	320 ± 20
MIP-1a	23 ± 4.6	22 ± 4.4	24 ± 4.5	24 ± 5.5	24 ± 4.5	25 ± 5.8
MIP-1B	23 ± 1.6	24 ± 1.4	22 ± 1.4	27 ± 1.7	**23 ± 2.0** ^b^	23 ± 1.6
RANTES	3,400 ± 580	3,100 ± 480	**4,200 ± 790** ^a^ ^,^ ^b^	3,500 ± 640	3,700 ± 570	3,700 ± 580
EOTAXIN	540 ± 46	540 ± 47	510 ± 49	510 ± 47	480 ± 46	520 ± 43
IP-10	290 ± 25	300 ± 26	**250 ± 28** ^a^ ^,^ ^b^	310 ± 32	250 ± 32 ^b^	320 ± 38
MIG/CXCL9	3,300 ± 300	3,600 ± 320	3,500 ± 310	3,200 ± 230	**2,900 ± 190** ^b^	3,000 ± 250
(Anti-Cancer)						
IFNa2	30 ± 5.6	29 ± 3.1	33 ± 4.7	35 ± 7.9	33 ± 5.7	37 ± 9.3
TNFB	58 ± 25	59 ± 24	66 ± 25	65 ± 26	67 ± 26	67 ± 25

^a^ p<0.05 compared to BDC.

^b^ p<0.05 p.m., compared to AM (same day).

Bold = statistically siginficant, p < 0.05.

Red blood cell parameters were also responsive to the altered atmosphere exposure. At MD3 a.m., the mild hypoxia induced an increase in mean corpuscular hemoglobin concentration (MCHC) and red cell distribution width (RDW) but a decrease in mean corpuscular volume (MCV) when compared to BDC (Table 2). The hyperoxic EVA recovered the MCV but an increase continued through to MD10. By MD7, increases in the RBC, hemoglobin and hematocrit were evident, and these alterations persisted through MD10 (Table 2). Platelet counts were generally stable throughout the mission, but achieved a statistically significant increase on MD10.

‘Fine’ leukocyte subsets were assessed, positively identified by surface marker expression, by flow cytometry analysis. Although mostly unaltered through the MD3 a.m. sampling, broad alterations were evident following the hyperoxic exposures. These included increases in total B cells and decreases in NK and NKT percentages (Table 2). A significant decrease in the cytotoxic T cell subset was evident following MD3 hyperoxic EVA, but this reversed and increased following MD7 hyperoxic EVA. No parallel changes in the helper T percentage was detected (Table 2). An increase in the maturation state of the helper-T population was evident with shifts towards CD4^+^ Effector Memory cells. However, this was not detected in the cytotoxic subset, where a significant increase in the ‘naïve’ subset was observed (Table 2).

### Plasma cytokine concentrations

No significant alterations were observed following the initial period of hypoxia exposure (MD3 a.m. vs BDC). Following the MD3 hyperoxic EVA, significant alterations were observed in the plasma concentration of several cytokines: IL-6 (↑), IL-12p40 (↑), IL-18 (↑), IL17E (↓), IL-17A (↑), G-CSF (↑), IL-15 (↑), M-CSF (↓), MCP-1 (↑), RANTES (↑) and IP-10 (↓) (Table 3). By the AM sample of MD7, IL-18, IL-1RA, EGF, PDGF-AA and PDGF-AB/BB were significantly elevated compared to BDC (Table 3). Following the MD7 hyperoxic EVA, the plasma concentration of several cytokines decreased compared to MD7 a.m. timepoint: EGF, PDGF-AA, PDGF-AB/BB, M-CSF, MIP-1B, IP-10, and MIG/CXCL9 (Table 3). Only two cytokines were consistent between the two EVA mission days with significant decreases seen in M-CSF and IP-10. At the AM sampling on MD10, 9 of the 10 categorized inflammatory cytokines were elevated, however only IL-22 was significantly elevated (Table 3). IL-1ra was also elevated at MD10a.m., however, this was not statistically significant.

**TABLE 3:** Leukocyte function assessed via mitogen stimulated (48h culture) cytokine profiles (pg/ml supernatant) (data are means ± SEM).

Variable	BDC AM	MD3 a.m.	MD3 p.m.	MD7 a.m.	MD7 p.m.	MD10 a.m.
(anti CD3^+^CD28 stimulation)						
GM-CSF	58 ± 8.1	43 ± 7.0	48 ± 6.3	48 ± 8.8	40 ± 6.4	42 ± 6.3
IFNy	3,100 ± 830	2,300 ± 730	3,000 ± 730	3,000 ± 1,000	3,900 ± 1,200	2,100 ± 510
IL-10	530 ± 84	530 ± 110	**860 ± 200** ^a^ ^,^ ^b^	640 ± 140	730 ± 190	530 ± 120
IL-12 (p70)	4.1 ± 0.83	3.2 ± 0.76	5.3 ± 1.7	4.1 ± 1.1	**6.1 ± 1.6** ^b^	3.6 ± 0.84
IL-13	53 ± 10	56 ± 18	73 ± 14	47 ± 11	46 ± 9.4	39 ± 6.8
IL-1B	23 ± 3.2	36 ± 18	31 ± 4.7	20 ± 3.5	**57 ± 11** ^a^ ^,^ ^b^	25 ± 3.8
IL-2	20 ± 2.4	27 ± 5.6	27 ± 4.2	20 ± 2.8	**29 ± 4.4** ^b^	19 ± 2.9
IL-4	43 ± 6.7	48 ± 12	55 ± 9.1	42 ± 7.7	49 ± 10	37 ± 7.2
IL-5	16 ± 3.2	18 ± 6.0	27 ± 7.4	17 ± 4.4	16 ± 3.6	13 ± 2.7
IL-6	61 ± 11	86 ± 33	**110 ± 23** ^a^	52 ± 13	**350 ± 110** ^a^ ^,^ ^b^	76 ± 24
IL-7	33 ± 6.4	33 ± 7.0	34 ± 7.1	33 ± 5.7	**40 ± 8.1** ^b^	40 ± 8.0
IL-8	9,500 ± 1,100	8,300 ± 1,500	**12000 ± 1700** ^b^	9,000 ± 1,500	**14000 ± 2000** ^b^	6,600 ± 1,300
TNFa	430 ± 52	310 ± 55	390 ± 57	410 ± 71	380 ± 50	**290 ± 43** ^a^
(PMA + ionomycin stimulation)						
GM-CSF	5,700 ± 340	5,300 ± 440	**6,200 ± 410** ^b^	5,300 ± 340	5,500 ± 430	**4,900 ± 370** ^a^
IFNy	Beyond linearity					
IL-10	1,000 ± 120	950 ± 68	**1800 ± 200** ^a^ ^,^ ^b^	**1,300 ± 150** ^a^	**1,600 ± 240** ^a^	1,100 ± 120
IL-12 (p70)	22 ± 3.7	21 ± 3.3	17 ± 2.5	21 ± 3.2	24 ± 3.4	22 ± 3.0
IL-13	1,400 ± 150	1,400 ± 150	**2,300 ± 240** ^a^ ^,^ ^b^	1,500 ± 200	**2,100 ± 200** ^a^ ^,^ ^b^	1,500 ± 150
IL-1B	110 ± 8.5	110 ± 13	130 ± 15	280 ± 120	280 ± 150	250 ± 57
IL-2	31000 ± 1800	30000 ± 1,600	**34000 ± 1,600** ^b^	32000 ± 2000	32000 ± 2,400	29000 ± 1900
IL-4	1,200 ± 100	1,100 ± 91	**1800 ± 150** ^a^ ^,^ ^b^	1,200 ± 99	**1,600 ± 160** ^a^ ^,^ ^b^	1,100 ± 110
IL-5	310 ± 36	310 ± 34	**550 ± 69** ^a^ ^,^ ^b^	320 ± 27	**460 ± 53** ^a^ ^,^ ^b^	300 ± 31
IL-6	1,500 ± 120	1,400 ± 110	**2,200 ± 220** ^a^ ^,^ ^b^	1,400 ± 100	**2,200 ± 210** ^a^ ^,^ ^b^	1,500 ± 98
IL-7	42 ± 7.2	45 ± 7.5	43 ± 7.1	51 ± 8.1^a^	50 ± 8.4	47 ± 7.2
IL-8	Beyond linearity					
TNFa	5,800 ± 420	5,600 ± 370	**7,700 ± 650** ^a^ ^,^ ^b^	**6,600 ± 410** ^a^	6,600 ± 430	**5,000 ± 380** ^a^
(LPS stimulation)						
GM-CSF	50 ± 5.9	47 ± 6.9	**65 ± 7.0** ^b^	43 ± 4.5	**66 ± 6.2** ^b^	42 ± 3.5
IFNy	7,200 ± 1,500	8,900 ± 1700	**18000 ± 1700** ^a^ ^,^ ^b^	5,700 ± 880	**16000 ± 1700** ^a^ ^,^ ^b^	10000 ± 1,300
IL-10	3,500 ± 350	2,700 ± 400^a^	**5,700 ± 1,000** ^a^ ^,^ ^b^	3,800 ± 610	**4,400 ± 740** ^b^	**2,800 ± 400** ^a^
IL-12 (p70)	14 ± 1.6	23 ± 3.9^a^	**46 ± 6.9** ^a^ ^,^ ^b^	18 ± 4.2	**36 ± 7.3** ^a^ ^,^ ^b^	**29 ± 5.2** ^a^
IL-13	13 ± 2.5	12 ± 2.2	**17 ± 2.1** ^b^	9.8 ± 1.1	**13 ± 1.3** ^b^	11 ± 1.3
IL-1B	5,200 ± 890	4,800 ± 660	**11000 ± 1,300** ^a^ ^,^ ^b^	**6,300 ± 850** ^a^	**7,200 ± 850** ^a^ ^,^ ^b^	**7,300 ± 750** ^a^
IL-2	12 ± 1.7	9.9 ± 1.9	**16 ± 1.7** ^b^	16 ± 1.4	17 ± 1.7	14 ± 1.3
IL-4	26 ± 1.9	27 ± 2.9	**34 ± 3.7** ^a^ ^,^ ^b^	25 ± 1.9	**27 ± 2.6** ^b^	26 ± 2.4
IL-5	4.4 ± 0.29	4.1 ± 0.30	**5.1 ± 0.29** ^b^	4.3 ± 0.36	4.7 ± 0.37	4.5 ± 0.26
IL-6	7,900 ± 580	7,600 ± 790	**11000 ± 780** ^a^ ^,^ ^b^	9,000 ± 770	**9,700 ± 750** ^a^ ^,^ ^b^	**9,300 ± 790** ^a^
IL-7	41 ± 6.9	43 ± 7.4	46 ± 8.2	47 ± 7.1	48 ± 7.9	49 ± 7.6
IL-8	Beyond linearity					
TNFa	4,800 ± 470	4,300 ± 440	**7,700 ± 880** ^a^ ^,^ ^b^	5,200 ± 650	**6,900 ± 960** ^a^ ^,^ ^b^	5,300 ± 460

^a^p < 0.05 compared to BDC.

^b^p < 0.05 p.m., compared to AM (same day).

Bold = statistically siginficant, p < 0.05.

### Leukocyte function/mitogen stimulated cytokine profiles

Supernatant cytokine concentrations following mitogenic stimulation of whole blood cells was used as a functional assessment of immunocytes. Different mitogens allowed a snapshot of the functional capacity of different leukocyte subsets. Following stimulation with all mitogens, no functional alterations were observed following the initial exposure to mild hypoxia (MD3 a.m.). Following the MD3 hyperoxic EVA, stimulation with antibodies to CD3 and CD28, triggered a significant increase in IL-10, IL-6 and IL-8 (Table 4). No alterations were evident in the MD7 a.m. sample, but after the MD7 EVA, increases were detected for six cytokines, with IL-8 consistently elevated across both EVAs (Table 4). On the morning of MD10, no alterations were observed compared to baseline sans a decrease in TNFa.

**TABLE 4:** Leukocyte function assessed via mitogen stimulated (48 h culture) cytokine profiles (pg/ml supernatant).

Variable	BDC AM	MD3 AM	MD3 PM	MD7 AM	MD7 PM	MD10 AM
(anti CD3^+^CD28 stimulation)						
GM-CSF	58 ± 8.1	43 ± 7.0	48 ± 6.3	48 ± 8.8	40 ± 6.4	42 ± 6.3
IFNy	3,100 ± 830	2,300 ± 730	3,000 ± 730	3,000 ± 1,000	3,900 ± 1,200	2,100 ± 510
IL-10	530 ± 84	530 ± 110	**860 ± 200** ^a^ ^,^ ^b^	640 ± 140	730 ± 190	530 ± 120
IL-12 (p70)	4.1 ± 0.83	3.2 ± 0.76	5.3 ± 1.7	4.1 ± 1.1	**6.1 ± 1.6** ^b^	3.6 ± 0.84
IL-13	53 ± 10	56 ± 18	73 ± 14	47 ± 11	46 ± 9.4	39 ± 6.8
IL-1B	23 ± 3.2	36 ± 18	31 ± 4.7	20 ± 3.5	**57 ± 11** ^a^ ^,^ ^b^	25 ± 3.8
IL-2	20 ± 2.4	27 ± 5.6	27 ± 4.2	20 ± 2.8	**29 ± 4.4** ^b^	19 ± 2.9
IL-4	43 ± 6.7	48 ± 12	55 ± 9.1	42 ± 7.7	49 ± 10	37 ± 7.2
IL-5	16 ± 3.2	18 ± 6.0	27 ± 7.4	17 ± 4.4	16 ± 3.6	13 ± 2.7
IL-6	61 ± 11	86 ± 33	**110 ± 23** ^a^	52 ± 13	**350 ± 110** ^a^ ^,^ ^b^	76 ± 24
IL-7	33 ± 6.4	33 ± 7.0	34 ± 7.1	33 ± 5.7	**40 ± 8.1** ^b^	40 ± 8.0
IL-8	9,500 ± 1,100	8,300 ± 1,500	**12000 ± 1700** ^b^	9,000 ± 1,500	**14000 ± 2000** ^b^	6,600 ± 1,300
TNFa	430 ± 52	310 ± 55	390 ± 57	410 ± 71	380 ± 50	**290 ± 43** ^a^
(PMA + ionomycin stimulation)						
GM-CSF	5,700 ± 340	5,300 ± 440	**6,200 ± 410** ^b^	5,300 ± 340	5,500 ± 430	**4,900 ± 370** ^a^
IFNy	Beyond linearity					
IL-10	1,000 ± 120	950 ± 68	**1800 ± 200** ^a^ ^,^ ^b^	**1,300 ± 150** ^a^	**1,600 ± 240** ^a^	1,100 ± 120
IL-12 (p70)	22 ± 3.7	21 ± 3.3	17 ± 2.5	21 ± 3.2	24 ± 3.4	22 ± 3.0
IL-13	1,400 ± 150	1,400 ± 150	**2,300 ± 240** ^a^ ^,^ ^b^	1,500 ± 200	**2,100 ± 200** ^a^ ^,^ ^b^	1,500 ± 150
IL-1B	110 ± 8.5	110 ± 13	130 ± 15	280 ± 120	280 ± 150	250 ± 57
IL-2	31000 ± 1800	30000 ± 1,600	**34000 ± 1,600** ^b^	32000 ± 2000	32000 ± 2,400	29000 ± 1900
IL-4	1,200 ± 100	1,100 ± 91	**1800 ± 150** ^a^ ^,^ ^b^	1,200 ± 99	**1,600 ± 160** ^a^ ^,^ ^b^	1,100 ± 110
IL-5	310 ± 36	310 ± 34	**550 ± 69** ^a^ ^,^ ^b^	320 ± 27	**460 ± 53** ^a^ ^,^ ^b^	300 ± 31
IL-6	1,500 ± 120	1,400 ± 110	**2,200 ± 220** ^a^ ^,^ ^b^	1,400 ± 100	**2,200 ± 210** ^a^ ^,^ ^b^	1,500 ± 98
IL-7	42 ± 7.2	45 ± 7.5	43 ± 7.1	51 ± 8.1 ^a^	50 ± 8.4	47 ± 7.2
IL-8	Beyond linearity					
TNFa	5,800 ± 420	5,600 ± 370	**7,700 ± 650** ^a^ ^,^ ^b^	**6,600 ± 410** ^a^	6,600 ± 430	**5,000 ± 380** ^a^
(LPS stimulation)						
GM-CSF	50 ± 5.9	47 ± 6.9	**65 ± 7.0** ^b^	43 ± 4.5	**66 ± 6.2** ^b^	42 ± 3.5
IFNy	7,200 ± 1,500	8,900 ± 1700	**18000 ± 1700** ^a^ ^,^ ^b^	5,700 ± 880	**16000 ± 1700** ^a^ ^,^ ^b^	10000 ± 1,300
IL-10	3,500 ± 350	2,700 ± 400 ^a^	**5,700 ± 1,000** ^a^ ^,^ ^b^	3,800 ± 610	**4,400 ± 740** ^b^	**2,800 ± 400** ^a^
IL-12 (p70)	14 ± 1.6	23 ± 3.9 ^a^	**46 ± 6.9** ^a^ ^,^ ^b^	18 ± 4.2	**36 ± 7.3** ^a^ ^,^ ^b^	**29 ± 5.2** ^a^
IL-13	13 ± 2.5	12 ± 2.2	**17 ± 2.1** ^b^	9.8 ± 1.1	**13 ± 1.3** ^b^	11 ± 1.3
IL-1B	5,200 ± 890	4,800 ± 660	**11000 ± 1,300** ^a^ ^,^ ^b^	**6,300 ± 850** ^a^	**7,200 ± 850** ^a^ ^,^ ^b^	**7,300 ± 750** ^a^
IL-2	12 ± 1.7	9.9 ± 1.9	**16 ± 1.7** ^b^	16 ± 1.4	17 ± 1.7	14 ± 1.3
IL-4	26 ± 1.9	27 ± 2.9	**34 ± 3.7** ^a^ ^,^ ^b^	25 ± 1.9	**27 ± 2.6** ^b^	26 ± 2.4
IL-5	4.4 ± 0.29	4.1 ± 0.30	**5.1 ± 0.29** ^b^	4.3 ± 0.36	4.7 ± 0.37	4.5 ± 0.26
IL-6	7,900 ± 580	7,600 ± 790	**11000 ± 780** ^a^ ^,^ ^b^	9,000 ± 770	**9,700 ± 750** ^a^ ^,^ ^b^	**9,300 ± 790** ^a^
IL-7	41 ± 6.9	43 ± 7.4	46 ± 8.2	47 ± 7.1	48 ± 7.9	49 ± 7.6
IL-8	Beyond linearity					
TNFa	4,800 ± 470	4,300 ± 440	**7,700 ± 880** ^a^ ^,^ ^b^	5,200 ± 650	**6,900 ± 960** ^a^ ^,^ ^b^	5,300 ± 460

^a^ p < 0.05 compared to BDC.

^b^ p < 0.05 p.m., compared to AM (same day).

Bold = statistically siginficant, p < 0.05.

Stimulation with PMA and ionomycin is a broader, more powerful pharmacological stimulus that bypasses intracellular signaling pathways. It acts on all the leukocyte subsets. Following PMA/I stimulation, no alterations were observed in the MD3 a.m. sampling, but following the MD3 EVA increases were observed in 8 different cytokines (Table 4). Similarly, after the MD7 a.m. sampling there was only a lingering increase in IL-10, IL-7, and TNFa compared to BDC, but following the MD7 EVA sampling there was a significant increase in four different cytokines (IL-13, IL-4, IL-5, and IL-6) compared to MD7 a.m. (Table 4). These four cytokines were significantly increased after both hyperoxic EVA missions days. On the morning of MD10 both GM-CSF and TNFa were reduced compared to the BDC timepoint.

Stimulation with LPS is a targeted stimulus that primarily activates monocytes via their CD14 cell surface receptor. Following LPS stimulation, no alterations were observed in the MD3 a.m. sampling, but following the MD3 EVA (timepoint MD3 p.m.) significant increases were observed in 11 of the 12 measurable cytokines when compared to MD3 a.m. (Table 4). Similarly, after the MD7 a.m. sampling only IL-1b was increased when compared to baseline, but following the MD7 EVA sampling there was a significant increase in 9 of the 12 measurable cytokines when compared to MD7 a.m. (Table 4). Only IL-2 and IL-5 did not respond in the same manner to both EVA mission days. On the morning of MD10, IL-10 was significantly decreased while IL-12 (p70), IL-1B, and IL-6 were significantly increased compared to BDC.

### Saliva cortisol

Mean saliva cortisol was not elevated across any of the 11 study days (Table 5). Values ranged from 0.39 to 0.52 µg/dL.

**TABLE 5:** Saliva Cortisol (µg/dL), Latent Herpesvirus DNA (copies/mL saliva), incidence per 3 missions; (n = 23).

Variable	BDC	MD1	MD2	MD3	MD4	MD5	MD6	MD7	MD8	MD9	MD10	MD11
Mean saliva cortisol concentration (AM - µg/dL)												
Saliva Cortisol	0.42	0.41	0.51	0.45	0.41	0.49	0.39	0.41	0.46	0.49	0.52	0.49
Mean latent virus reactivation (copies/mL)												
Saliva EBV	42161	18868	8,550	26121	12313	16473	17148	12993	19267	15267	10001	16771
Saliva HSV1	0	0	0	5,342	678917	977	0	0	0	617	169123	44255
Saliva VZV	0	0	0	0	0	0	3	0	0	0	0	0
Urine CMV	0	0	0	0	0	0	0	0	0	0	0	0
Latent virus reactivation - Percent Incidence across 3 missions/study day; n = 23												
Saliva EBV	43%	35%	48%	57%	43%	26%	43%	35%	48%	43%	35%	39%
Saliva HSV1	0%	0%	0%	4%	4%	4%	0%	0%	0%	4%	4%	4%
Saliva VZV	0%	0%	0%	0%	0%	0%	4%	0%	0%	0%	0%	0%
Urine CMV	0%	0%	0%	0%	0%	0%	0%	0%	0%	0%	0%	0%

### Saliva/urine herpesvirus DNA

Saliva was collected first thing in the morning at BDC, and each of the 11 mission days. No shedding of CMV, in urine, was detected across any of the study days. Only a single positive sample was observed for saliva VZV, and only 3 copies/mL (Table 5). HSV DNA in saliva was observed repeatedly in a single subject, on MD3-5 and MD9-11 (Table 5). EBV reactivation in saliva was observed in more subjects, ranging from 25% to 54% positive samples from the total 23 subject count, however no statistically significant increase was observed in any of the 11 mission days, compared to baseline (Table 5).

## Discussion

There are reports that hypoxia may have benefit as a prophylactic, or therapeutic, for some disease states, and that the addition of intermittent hyperoxia during the exposures may heighten the magnitude of benefit (Uzun et al., 2023). A mechanism of action may be the upregulation of reactive oxygen species and hypoxia-inducible genes. Other reports suggest that hyperoxia exposures are well tolerated (Netzer et al., 2024; Walker et al., 2018). There is, however, no broad evidence base informative of the effect of routine cycling between hypoxia and hyperoxia on aspects physiology. The current study was conducted at the Johnson Space Center, to assess the condition of living in a habitat at mild hypoxia, with periodic decompression with hyperoxic EVA activity simulation (atmospheres being defined by vehicle and mission constraints), on multiple aspects of physiology. The goal was to ascertain if the atmospheric cycling increased any specific clinical risks. The immune assessment was part of that parent study, and utilized a battery of assays previously validated to monitor multiple aspects of immune dysregulation observed in astronauts during spaceflight. Blood samples were collected as described in the schedule above, timed to specifically assess the effect of mild hypoxia, hyperoxic EVA activity, decompression stress, and alternating between the two environments. These assays include peripheral leukocyte distribution as a measure of *in-vivo* mobilization of immune subsets generally related to immune responses, plasma cytokine concentrations as an indicator of the *in-vivo* status of the hormonal regulation of immunity between particular immune ‘biases’, and immune cell functional capacity assessed following mitogenic stimulation. Additionally, saliva stress hormone concentrations and the subclinical reactivation of latent herpesviruses were monitored daily.

The data revealed minimal alterations, immune, stress, etc., resulting from the mildly hypoxic cabin environment, primarily associated with the MD3 a.m. sampling (Table 1). At this point, the subjects had experienced almost 3 continuous days in the exploration atmosphere environment. On the surface, this would seem somewhat discordant with our previous finding of profound immune sensitization and latent virus reactivation while living at Concordia Station, Antarctica. These results, as they themselves were somewhat polar opposite from the spaceflight findings, were generally attributed to the confounding variable of persistent hypobaric hypoxia. However, it is relevant that the hypoxia experienced at Concordia Station is profound, with an atmosphere pp O2 of 101.6 mmHg (with an ambient pressure of 522 mmHg), as compared to 159 mmHg at sea level (760 mmHg ambient). This chamber study was NOT designed to assess impactful hypoxia, and was only adjusted to 141–144 mmHg pp O2 but in a significantly lower pressure than Concordia (422/493 mmHg ambient), to coincide with the planned deep space atmosphere. The authors believe this is why noteworthy effects of hypoxia, despite a more pronounced hypobaric stress, were not observed in this chamber study.

The post-hyperoxic EVA findings however, primarily MD3 and MD7 p.m. samplings, were consistent. A redistribution of leukocytes was evident, primarily an increase in neutrophils, with a relative decrease in lymphocytes, monocytes, basophils and eosinophils (Table 2). The RBC, hematocrit and hemoglobin were all increased, and RBC morphology was altered, with an increase in corpuscular volume and hemoglobin (Table 2). Lymphocyte subsets were also altered post EVA, with an increase in the T cell percentage, a reduction in cytotoxic T cells (without a corresponding increase in helper T cells), and an increase in the maturation state of the helper T cell subsets, but a converse reduction in the maturation state of the cytotoxic compartment (Table 2). B cell subsets also moved towards a increased maturation state. Plasma cytokine dysregulation was also evident post- EVA, with an increase in the concentration of several cytokines associated with inflammation (IL-6, IL-12, IL-18) and chemokines also associated with inflammation (MCP-1, IP-10) (Table 3). By MD7, IL-1ra was also increased, confirming a pro-inflammatory state in the subjects. Alterations were also observed for several other cytokines, including RANTES, PDGF, and others, that likely point to disruption of other immunoregulatory processes (Table 3). Minimal alterations in plasma cytokines were observed by MD10, indicating that the previous alterations were indeed associated only with the hyperoxic EVA exposures and not the cabin hypoxia. Leukocyte function, as determined by supernatant cytokine production following blood leukocyte mitogenic stimulation in culture (48 h) was dramatically altered post EVA exposure (Table 4). Increases in multiple cytokines were observed after the EVA activities on MD3 and MD7, with far less alterations at the ‘cycling’ timepoints on the mornings of MD7 and MD10. These results confirm a ‘sensitization’ or increase in immune function was evident, moreso seemingly in the innate populations (LPS stimulation) and broadly (PMA-I stimulation), than was observed in following the T cell specific stimulation (anti-3/28) (Table 4).

**FIGURE 4:**
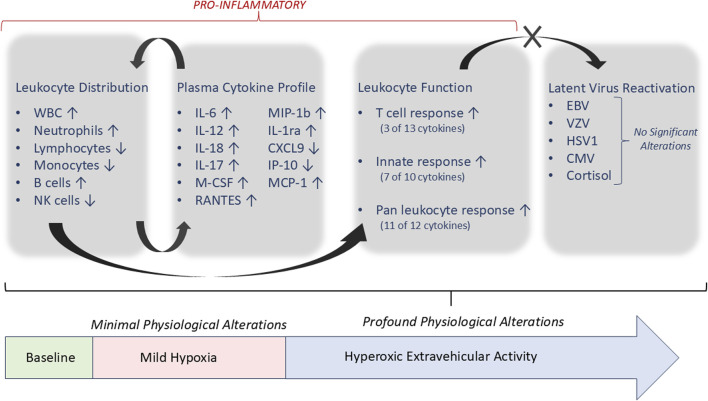
Summary of significant alterations associated with hyperoxic EVA activity, and immunological associations. All parameters listed with ‘↑/↓’ were significantly altered as described, compared to baseline, except where noted.

The primary finding from this study is that, while living in a mildly hypoxic exploration atmosphere did not have a profound impact on immunity, the participation in hypobaric/hyperoxic EVA activities impacted numerous and varied immune parameters. A summation of the primary significant impacts of hyperoxic EVA are represented in Figure 4. This study was designed to be an applied, clinical research assessment, that monitored four basic categories of outputs: (A) leukocyte distribution, (B) plasma cytokine concentrations, (C) leukocyte function, and (D) latent virus reactivation. We have previously discussed how these parameters generally provide a good snapshot of immune mobilization (A), immunological response biases (B), environmental impacts on function (C), and an adverse clinical outcome that may be quantitated (D). This is insufficient for a detailed biological pathway analysis, however is sufficient for a broad assessment of subject immune function, determining the nature of any dysregulation, and projections of clinical risk. The significant findings confirm immune mobilization of primarily innate cells, a generally pro-inflammatory sensitization, and profound environmental impacts in reducing leukocyte function, without a concurrent rise in latent virus reactivation as is commonly observed in astronauts (Figure 4). Most of the cytokines that were increased in the subject’s plasma, although not to a profound level, are indeed associated with inflammation. The reductions in cell function were most remarkable in both the consistency across the 13 cytokines measures, and across the 3 distinct stimuli. PMA and ionomycin stimulates the nucleus directly and bypass the intracellular signaling pathways required for activation *in-vivo*, yet it yielded the most consistent reduction in function (11/12 cytokines). In previous stress-analog studies a reduction in cellular function is almost always associated with both stress and the reactivation of latent herpesviruses. The most likely reason for the absence of latent virus reactivation in this study, and the unchanging cortisol levels, seems to be the relatively short duration of the chamber habitation, and the intermittent ‘rest’ days allowing a degree of recovery, something that does not happen during spaceflight of any duration.

It should be noted that although stimulations during cell culture were utilized to assess immunocyte functional capacity, these cultures occurred during normoxic and normobaric laboratory conditions. An excellent indicator of direct hypoxia/hyperoxia effects on cell function would be inclusion of altered-atmosphere cell culture. In such a study, alterations may be ‘induced’ by the altered atmosphere culture conditions, even in healthy individuals, essentially creating an artifactual dysregulation that is not representative of the *in-vivo* condition. That was not the goal of this study. Instead, the goal was to assess dysregulation induced *in-vivo*, by performing ‘normal’ stimulations *ex-vivo* and observing functional decrements. This is analogous to spaceflight investigations of astronauts with cellular function altered by microgravity. Dysregulation may be induced in healthy subjects’ cells by culturing in modeled-microgravity conditions, in a bioreactor or clinostat device. For functional assessment of astronauts during spaceflight however, blood samples are collected onboard ISS, and returned for terrestrial ‘static’ (normal, 1xG) cell culture to monitor function. While altered culture conditions are informative for basic cell biology studies, to assess an individual’s immune function in a clinical fashion is not desirous to induce artifactual decrements by culturing in unusual conditions.

Although the changes described above associated with hyperoxic simulated EVAs are noteworthy, they are generally associated with mobilization, inflammation, and cell sensitization. We are therefore unsurprised that in only an 11 days study, there was no increase in stress hormones or any clinically relevant reactivation of latent herpesviruses. Such reactivation, as is commonly observed during spaceflight, is generally associated with immune suppression, and is commonly observed in astronauts during spaceflight (Rooney et al., 2019). Immune suppression was not observed during this study. Nor does this study represent the creation of a true ground-based Space Flight analog in terms of immune response. The goal was not to recreate mission stressors but to create a high-fidelity situation to assess the effect of altered atmospheres in parallel with the physical activity of EVA.

The results from this study confirm previous observations that altered oxygen partial pressures in the atmosphere can profoundly influence immunity. Limitations may include an imperfect analog relevance to spaceflight, as other mission associated stressor (microgravity, radiation, etc.) are absent. Generally, the literature regarding hypoxia exposure is derived from assessments of individuals living at altitude, and the findings generally indicate that such hypoxia exposure is immune sensitizing (Kubo et al., 1998; Hartmann et al., 2000; Coussons-Read et al., 2002; Facco et al., 2005; Ermolao et al., 2009). The previous study at Concordia station confirmed this scenario but using a more sophisticated panel of assays as defined by immune alterations in astronauts (Feuerecker et al., 2014; Feuerecker et al., 2019). The effect of very mild hypoxia in conjunction with intermittent hyperoxic EVA on immunity was unknown. Decompression stress is also known to trigger an inflammatory response. Particularly following hyperbaric (vs hypobaric) exposures, decompression also causes elevation of inflammatory markers even without clinical signs or symptoms of decompression illness (Mitchell, 2024; Mrakic-Sposta et al., 2024). Although similar studies in hypobaric exposures are sparse, blood markers for hypobaric exposures show elevation of inflammatory markers (Connolly et al., 2023). The data seem to indicate that the mild hypoxia condition, even less than hypoxia experienced in an equivalent altitude of Denver, is not disruptive. The hyperoxia and decompression stress of EVAs result in a profound mobilization inflammation and sensitization of immunity as clearly evident in the samples taken after the simulated EVAs (PM). Humans during deep space missions will be a complicated applied experiment consisting of multiple mission stressors and influencing variables, including microgravity stress circadian misalignment, altered diet, and all this in a much more limited habitable volume with communication delays. Altered atmosphere simply adds yet another variable that can influence human physiology. We suggest further characterization is warranted as scenarios with different atmospheres are tested with more EVAs, as well as an ability to monitor immunity and crew members during deep space missions. Having a better understanding of immune changes during atmospheric cycling will allow for development of broad biomedical countermeasures capable of restoring immune function.

## Data Availability

The datasets presented in this study can be found in online repositories. The names of the repository/repositories and accession number(s) can be found below: Data available at the NASA Life Sciences Data Archive (LSDA) upon request.
